# The I. H. A. and Its Supposed Shortcomings

**Published:** 1884-02

**Authors:** E. W. Berridge

**Affiliations:** Member and Corresponding Secretary of the I. H. A.; London


### THE I. H. A. AND ITS SUPPOSED SHORTCOMINGS.

DEAR EDITOR:—In your note you call my attention to your editorial in the December number of THE HOMŒOPATHIC PHYSICIAN, and express a hope that I should indorse it. I have read it, but I cannot altogether do so. In the first place, you bring this charge against the I. H. A. that its "innumerable resolutions" are "mostly useless, and often very silly," and that its clinical cases have been "useful and valuable to a certain extent, but they present little that is new." Now, with regard to the resolutions passed since the Association was established in 1880, it happens that I am the proposer of the majority of them, and it is rather rough on me to ask me to indorse an opinion pronouncing my own work as "useless and very silly." Whether useless or not, time will show, but they were every one written with a special object. The only resolution to which you can seriously take exception was one (not mine) to the effect that THE HOMŒOPATHIC PHYSICIAN should no longer be the organ of the I. H. A. (September, 1882, page 374), and, as this resolution was not accepted by the Association, as being founded on a misconception, and as you say (December, 1883, page 369) that THE HOMŒOPATHIC PHYSICIAN is the organ of no Association, even this unfortunate resolution can hardly be justly objected to by you.

As for the clinical cases, I think that these, when accompanied by a description of the method whereby the remedy was selected in each case, and by illustrative comments, are among the most valuable papers that can be presented to the profession. To present something "new" would often be difficult, seeing that the symptoms, or at least those characteristic, will be already found in the materia medica. However, if "newness" is desired, let us all the next time report the most extraordinary cases we can find, something no one has ever seen before, and perhaps never will see again. I, for one, will report a case of nymphomania cured by *Lachesis* (selected according to Fincke's proving in *H. M.*, Vol. I), which is the most extraordinary case I ever heard of, and if you can prove that there is nothing new in it, I will undertake to eat my hat. But are you not unintentionally doing injustice to the I. H. A. by omitting notice of the other valuable essays? Surely Dr. Leila Rendell's proving of *Ammonium carbonicum* is a specimen of "industrious work."

Thus far I cannot indorse your editorial; but with the latter part I am in sympathy. You advise that the I. H. A. should publish standard works—to which I say, Amen. But I think it cannot be correctly compared to the *Hahnemannian Publishing Society*, for the latter is simply a publishing society which any one can join by paying a guinea subscription, while the former is an Association to which only Hahnemannians are admitted, and the annual subscription is a dollar. Hence, the question of money comes prominently into notice. Even Hering's *Guiding Symptoms* is wearily dragging its slow length along owing to want of proper pecuniary support. Yet, if your scheme can be carried out, by all means let it be done. Why should not certain works be published, if not by the I. H. A., at least under its auspices and with its indorsement? I am sure we shall be pleased as an Association to indorse your invaluable *Cough Repertory*, which is certainly the most complete of all ever published. Of one thing I am sure: whatever the I. H. A. publishes will be as *complete* as possible, thereby differing from the *imperfect* works which the *Hahnemannian Publishing Society* has mostly hitherto published. What the future work of this Society will be it is premature to conjecture. I trust it will be more complete than its early work; but this fact remains that some cures by *Kali bichromicum* which I recently sent to the compiler of this medicine were rejected because the CM potency was used (you shall have them when you have published what you still hold of mine), and my offer to arrange the symptoms of *Lac caninum*, daybooks, schema, cases, and comparisons was politely and sarcastically declined by Jupiter Maximus himself. So much for the fairness and liberality of *pretended* homœopaths; but it is only just the way in which the mongrel press has treated the Hahnemannians both here and elsewhere for many a year. We now shake off the dust of our feet against them, and leave them to—decay!

Thus far about the I. H. A.; and when the forthcoming volume of Transactions are published, I do not think any one need feel ashamed of them.

One more word about "Isopathy." I do not intend to say much, as my views have been already given and been—misunderstood! After my first paper in THE HOMŒOPATHIC PHYSICIAN, on "The Scientific Use of Nosodes," appeared, I received two strongly expressed letters from members of I. H. A., one censuring me for upholding Swan's supposed heresy, and the other blaming me in like vigorous style for injuring the reputation of the nosodes! But if critics will neglect the "totality of the symptoms," and base their remarks on a false "keynote," it is their fault, not mine.

In your December number Dr. Swan's letter is headed "Hahnemann the Founder of Isopathy," and your editorial "Hahnemann Not the Founder of Isopathy." As both assertions cannot be right, it is necessary to discover what is meant by Isopathy. If Isopathy means that *every* case of syphilis is to be treated and cured by potentized *Syphilinum*, then Hahnemann never taught it, neither did Swan indorse or discover it. But if Isopathy means that potentized *Syphilinum* will cure certain cases of syphilis when the symptoms correspond, then this is what Hahnemann taught and what Swan has indorsed and elaborated. The fact is, that Lux (according to Lippe) taught the former, which is a departure from Hahnemann; Swan never taught it, though his words have been misconstrued to mean it, and here he says that he simply claims just what Hahnemann claimed. It should not be forgotten that even diseases resulting from specific contagious poisons are very often complicated, and therefore to say that *Syphilinum* will cure uncomplicated syphilis is very different from saying that it will cure every case in which syphilis exists.

How then are we to discriminate? Simply by provings on the healthy organism, which Dr. Swan has done, both with *Syphilinum* and other nosodes. This leads me to your editorial reply. You say that Hahnemann, in the passage you quote, condemns the use of unproved remedies. No doubt he does condemn them, but the present instance is not a case in point, as he is not referring to "unproved remedies." On the contrary, by the use of the phrase, "not *sufficiently* ascertained," he necessarily implies that their pathogenetic effects had been partially ascertained, but not enough to base accurate prescriptions upon. Surely, the effects of syphilis upon a previously healthy person are as much a proving of crude *Syphilinum*, as a poisoning by *Aconite* is a proving of the same. And if we may unite in a pathogenesis a poisoning by *Aconite* and a proving with the CM potency, why not with *Syphilinum* or any other nosode? The only source of error, which *always* has to be guarded against, is the possible development of collateral symptoms from other sources; but repeated provings and clinical experience will soon eliminate these.

Now, as the I. H. A. is at present only an "ornamental" society, will not you and all the other members therefore set to *work* and—prove *Syphilinum* on yourselves; and then do the same with *Medorrhinum* and the other nosodes? I have tried to prove them, but can get no symptoms; they have, however, been proved by Dr. Swan himself, and by several other members of the I. H. A. And for the future, let us devote our combative energies to annihilating the mongrels instead of each other, more especially as the differences of opinion between the members of the I. H. A. are more apparent than real.

Yours fraternally,

E. W. BERRIDGE,

*Member and Corresponding Secretary of the I. H. A.*

